# Aqua­(furan-2-carboxyl­ato-κ*O*)(furan-2-carboxyl­ato-κ^2^ *O*,*O*′)(1,10-phenanthroline-κ^2^ *N*,*N*′)copper(II) methanol hemisolvate

**DOI:** 10.1107/S1600536809024428

**Published:** 2009-07-01

**Authors:** Yanfei Li, Junshan Sun, Shanghua Feng, Ruigang Xue, Jun Wang

**Affiliations:** a Department of Materials and Chemical Engineering, Taishan University, 271021 Taian, Shandong, People’s Republic of China

## Abstract

The asymmetric unit of the title compound, [Cu(C_5_H_3_O_3_)_2_(C_12_H_8_N_2_)_2_(H_2_O)]·0.5CH_3_OH, contains two Cu^II^ complex mol­ecules and one methanol solvent mol­ecule with the metal centres in strongly distorted octahedral coordination. The coordinated water mol­ecule is involved in inter­molecular O—H⋯O hydrogen bonding, which links the complex mol­ecules into chains propagating along the *c* axis. Neighbouring chains inter­act further *via* π–π inter­actions between the aromatic rings of 1,10-phenanthroline fragments [centroid–centroid distances = 3.726 (4) and 3.750 (4) Å].

## Related literature

For the crystal structures of related carboxyl­ate complexes with 1,10-phenanthroline, see: Ai *et al.* (2007 ▶); Li *et al.* (2007 ▶); Rodrigues (2004 ▶).
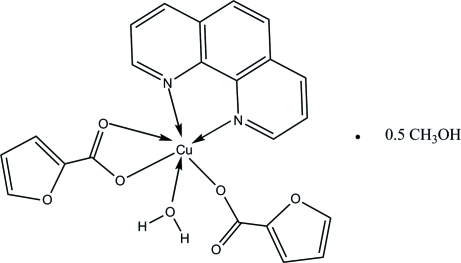


## Experimental

### 

#### Crystal data

[Cu(C_5_H_3_O_3_)_2_(C_12_H_8_N_2_)_2_(H_2_O)]·0.5CH_4_O
*M* *_r_* = 499.93
Tetragonal, 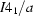

*a* = 34.129 (17) Å
*c* = 14.450 (6) Å
*V* = 16831 (14) Å^3^
*Z* = 32
Mo *K*α radiation
μ = 1.09 mm^−1^
*T* = 273 K
0.28 × 0.22 × 0.17 mm

#### Data collection

Bruker SMART APEX diffractometer
Absorption correction: multi-scan (*SADABS*; Sheldrick, 1996 ▶) *T*_min_ = 0.750, *T*_max_ = 0.836
43834 measured reflections
7448 independent reflections
4004 reflections with *I* > 2σ(*I*)
*R*_int_ = 0.100

#### Refinement

*R*[*F* ^2^ > 2σ(*F* ^2^)] = 0.055
*wR*(*F* ^2^) = 0.169
*S* = 0.97
7448 reflections
595 parameters
792 restraints
H-atom parameters constrained
Δρ_max_ = 0.60 e Å^−3^
Δρ_min_ = −0.54 e Å^−3^

### 

Data collection: *SMART* (Siemens, 1996 ▶); cell refinement: *SAINT* (Siemens, 1996 ▶); data reduction: *SAINT*; program(s) used to solve structure: *SHELXS97* (Sheldrick, 2008 ▶); program(s) used to refine structure: *SHELXL97* (Sheldrick, 2008 ▶); molecular graphics: *SHELXTL* (Sheldrick, 2008 ▶); software used to prepare material for publication: *SHELXTL*.

## Supplementary Material

Crystal structure: contains datablocks I, global. DOI: 10.1107/S1600536809024428/cv2568sup1.cif

Structure factors: contains datablocks I. DOI: 10.1107/S1600536809024428/cv2568Isup2.hkl

Additional supplementary materials:  crystallographic information; 3D view; checkCIF report

## Figures and Tables

**Table 1:** Hydrogen-bond geometry (Å, °)

*D*—H⋯*A*	*D*—H	H⋯*A*	*D*⋯*A*	*D*—H⋯*A*
O14—H14*B*⋯O5^i^	0.85	1.76	2.611 (8)	177
O14—H14*A*⋯O8	0.85	2.07	2.634 (7)	124
O13—H13*B*⋯O11	0.85	1.96	2.749 (7)	154
O13—H13*A*⋯O2	0.85	1.87	2.708 (7)	169
O15—H15⋯O4	0.82	1.86	2.474 (17)	131

Symmetry code: (i) 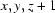.

**Fig. 1.**
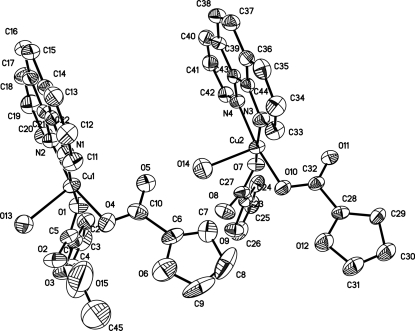
The content of asymmetric unit of the title compound, with atomic numbering and 30% probability displacement ellipsoids.

## supplementary crystallographic information

### Comment

Metal complexes with carboxylates are among the most investigated complexes in the field of coordination chemistry. In recent years, more and more attentions begin to be inclined to complexes with mixed-ligands such as 1,10-phenanthroline ligand (Ai *et al.*, 2007; Li *et al.*, 2007; Rodrigues, 2004). We selected a new carboxylic ligand with the cupric acetate in the presence of 1,10-phenanthroline co-ligand and obtained the title compound, (I).

In (I), the Cu centers exhibit a six-coordinated octahedron geometry with three O atoms from two carboxylic ligands[Cu—O 1.946 (4)–2.255 (4) Å] and one water molecule (Cu—O 1.937 (4)/%A) and two N atoms [Cu—N 2.011 (4), 2.023 (4)/%A] from 1,10-phenanthroline ligand. The crystal packing exhibits intra- and intermolecular  O—H···O hydrogen bonds (Table 1). The latter link the complex molecules into a one-dimensional infinite chain structure.

### Experimental

The reaction was carried out in 30 ml me thanol solvent. furan-2-carboxylic acid(0.224 g,2 mmol) and cupric acetate(0.199 g, 1 mmol) and 1,10-phenanthroline(0.180 g, 1 mmol) were mixed in the methanol solvent and stirred for 6 h. The resulting blue solution was filtered. The filtrate was placed for sevaral days yielding blue crystals.

The yield is 76% and elemental analysis: calc. for C_45_H_36_Cu_2_N_4_O_15_: C 54.05, H 3.63, N 5.60; found: C 54.32, H 3.39, N 5.22. The elemental analyses were performed with PERKIN ELMER MODEL 2400 SERIES II.

### Refinement

C-bound H atoms were placed in idealized positions, with C—H = 0.93/%A and *U*_iso_(H) = 1.2Ueq(C). O-bound H atoms were located in a difference Fourier map, but placed in idealized positions (O—H 0.82-0.85 Å) and refined as riding, with *U*_iso_(H) = 1.2Ueq(O).

### Crystal data

**Table:**

[Cu(C_5_H_3_O_3_)_2_(C_12_H_8_N_2_)(H_2_O)]·0.5CH_4_O	*D*_x_ = 1.578 Mg m^−^^3^
*M**_r_* = 499.93	Mo *K*α radiation, λ = 0.71073 Å
Tetragonal, *I*4_1_/*a*	Cell parameters from 2700 reflections
*a* = 34.129 (17) Å	θ = 2.3–17.6°
*c* = 14.450 (6) Å	µ = 1.09 mm^−^^1^
*V* = 16831 (14) Å^3^	*T* = 273 K
*Z* = 32	Block, blue
*F*(000) = 8192	0.28 × 0.22 × 0.17 mm

### Data collection

**Table:**

Bruker SMART APEX diffractometer	7448 independent reflections
Radiation source: fine-focus sealed tube	4004 reflections with *I* > 2σ(*I*)
graphite	*R*_int_ = 0.100
φ and ω scans	θ_max_ = 25.1°, θ_min_ = 1.2°
Absorption correction: multi-scan (*SADABS*; Sheldrick, 1996)	*h* = −24→40
*T*_min_ = 0.750, *T*_max_ = 0.836	*k* = −40→40
43834 measured reflections	*l* = −17→17

### Refinement

**Table:**

Refinement on *F*^2^	Primary atom site location: structure-invariant direct methods
Least-squares matrix: full	Secondary atom site location: difference Fourier map
*R*[*F*^2^ > 2σ(*F*^2^)] = 0.055	Hydrogen site location: inferred from neighbouring sites
*wR*(*F*^2^) = 0.169	H-atom parameters constrained
*S* = 0.97	*w* = 1/[σ^2^(*F*_o_^2^) + (0.0667*P*)^2^ + 46.5236*P*] where *P* = (*F*_o_^2^ + 2*F*_c_^2^)/3
7448 reflections	(Δ/σ)_max_ = 0.067
595 parameters	Δρ_max_ = 0.60 e Å^−^^3^
792 restraints	Δρ_min_ = −0.54 e Å^−^^3^

### Special details

**Table:**

Geometry. All e.s.d.'s (except the e.s.d. in the dihedral angle between two l.s. planes) are estimated using the full covariance matrix. The cell e.s.d.'s are taken into account individually in the estimation of e.s.d.'s in distances, angles and torsion angles; correlations between e.s.d.'s in cell parameters are only used when they are defined by crystal symmetry. An approximate (isotropic) treatment of cell e.s.d.'s is used for estimating e.s.d.'s involving l.s. planes.
Refinement. Refinement of *F*^2^ against ALL reflections. The weighted *R*-factor *wR* and goodness of fit *S* are based on *F*^2^, conventional *R*-factors *R* are based on *F*, with *F* set to zero for negative *F*^2^. The threshold expression of *F*^2^ > σ(*F*^2^) is used only for calculating *R*-factors(gt) *etc*. and is not relevant to the choice of reflections for refinement. *R*-factors based on *F*^2^ are statistically about twice as large as those based on *F*, and *R*- factors based on ALL data will be even larger.

### Fractional atomic coordinates and isotropic or equivalent isotropic displacement parameters (Å^2^)

**Table:**

	*x*	*y*	*z*	*U*_iso_*/*U*_eq_	
Cu1	0.41922 (2)	0.92317 (2)	0.34020 (6)	0.0567 (3)	
Cu2	0.40266 (2)	0.93097 (2)	0.83960 (5)	0.0545 (3)	
N1	0.47730 (13)	0.93179 (14)	0.3495 (3)	0.0526 (12)	
N2	0.41904 (14)	0.98143 (13)	0.3637 (3)	0.0537 (13)	
N3	0.46157 (13)	0.93354 (14)	0.8468 (4)	0.0584 (13)	
N4	0.40865 (13)	0.98941 (13)	0.8544 (3)	0.0522 (12)	
O1	0.36304 (12)	0.92475 (12)	0.3225 (3)	0.0682 (12)	
O2	0.34551 (14)	0.87386 (14)	0.4113 (4)	0.0920 (17)	
O3	0.26925 (14)	0.88894 (14)	0.3840 (4)	0.0917 (16)	
O4	0.42538 (14)	0.87018 (14)	0.2810 (4)	0.0790 (14)	
O5	0.42095 (15)	0.90712 (16)	0.1585 (4)	0.0922 (15)	
O6	0.42574 (16)	0.8048 (2)	0.1759 (4)	0.1119 (19)	
O7	0.34659 (11)	0.93629 (11)	0.8196 (3)	0.0611 (11)	
O8	0.32803 (13)	0.89471 (14)	0.9321 (4)	0.0826 (15)	
O9	0.25254 (13)	0.90035 (13)	0.8808 (4)	0.0809 (14)	
O10	0.40450 (12)	0.87529 (11)	0.8105 (3)	0.0622 (11)	
O11	0.41060 (14)	0.89599 (13)	0.6660 (3)	0.0758 (13)	
O12	0.41274 (16)	0.79989 (13)	0.7609 (4)	0.0909 (16)	
O13	0.41769 (13)	0.89163 (13)	0.4768 (3)	0.0825 (14)	
H13A	0.3946	0.8844	0.4631	0.099*	
H13B	0.4159	0.9007	0.5315	0.099*	
O14	0.39486 (14)	0.92340 (14)	0.9930 (4)	0.1002 (17)	
H14A	0.3857	0.9020	0.9716	0.120*	
H14B	0.4041	0.9177	1.0460	0.120*	
O15	0.4487 (5)	0.8142 (3)	0.3748 (11)	0.374 (11)	
H15	0.4304	0.8233	0.3450	0.561*	
C1	0.29690 (19)	0.91197 (19)	0.3408 (5)	0.0633 (17)	
C2	0.2805 (2)	0.9406 (2)	0.2934 (6)	0.081 (2)	
H2	0.2930	0.9600	0.2591	0.097*	
C3	0.2386 (2)	0.9356 (3)	0.3055 (6)	0.093 (2)	
H3	0.2187	0.9509	0.2803	0.112*	
C4	0.2345 (2)	0.9050 (3)	0.3605 (7)	0.099 (2)	
H4	0.2104	0.8955	0.3804	0.119*	
C5	0.33834 (19)	0.9016 (2)	0.3615 (5)	0.0671 (18)	
C6	0.42439 (19)	0.8399 (2)	0.1322 (7)	0.079 (2)	
C7	0.4270 (2)	0.8336 (2)	0.0467 (7)	0.091 (2)	
H7	0.4263	0.8539	0.0036	0.109*	
C8	0.4283 (2)	0.7958 (3)	0.0253 (8)	0.113 (3)	
H8	0.4302	0.7845	−0.0332	0.135*	
C9	0.4272 (3)	0.7777 (3)	0.1052 (9)	0.115 (3)	
H9	0.4276	0.7507	0.1123	0.138*	
C10	0.42390 (18)	0.8749 (2)	0.1990 (6)	0.0709 (18)	
C11	0.50628 (18)	0.90624 (19)	0.3447 (5)	0.0655 (17)	
H11	0.5001	0.8799	0.3368	0.079*	
C12	0.54568 (19)	0.9165 (2)	0.3516 (5)	0.0683 (18)	
H12	0.5651	0.8975	0.3487	0.082*	
C13	0.55537 (19)	0.9543 (2)	0.3620 (5)	0.0677 (17)	
H13	0.5816	0.9616	0.3648	0.081*	
C14	0.52599 (18)	0.98333 (19)	0.3684 (4)	0.0575 (15)	
C15	0.5328 (2)	1.0239 (2)	0.3838 (5)	0.0686 (17)	
H15A	0.5585	1.0329	0.3886	0.082*	
C16	0.5032 (2)	1.04922 (19)	0.3911 (5)	0.0698 (17)	
H16	0.5086	1.0756	0.4002	0.084*	
C17	0.46328 (19)	1.03649 (18)	0.3853 (4)	0.0603 (16)	
C18	0.4303 (2)	1.0611 (2)	0.3938 (5)	0.0731 (18)	
H18	0.4335	1.0877	0.4043	0.088*	
C19	0.3941 (2)	1.0457 (2)	0.3873 (5)	0.0776 (19)	
H19	0.3724	1.0620	0.3927	0.093*	
C20	0.3887 (2)	1.00561 (19)	0.3720 (5)	0.0690 (18)	
H20	0.3634	0.9956	0.3680	0.083*	
C21	0.45586 (17)	0.99693 (16)	0.3707 (4)	0.0504 (14)	
C22	0.48726 (17)	0.96997 (17)	0.3627 (4)	0.0493 (14)	
C23	0.28030 (18)	0.92319 (18)	0.8385 (5)	0.0574 (16)	
C24	0.26325 (19)	0.9482 (2)	0.7801 (5)	0.0709 (18)	
H24	0.2756	0.9667	0.7427	0.085*	
C25	0.2217 (2)	0.9409 (2)	0.7866 (6)	0.082 (2)	
H25	0.2017	0.9540	0.7555	0.099*	
C26	0.2175 (2)	0.9119 (2)	0.8455 (6)	0.084 (2)	
H26	0.1936	0.9006	0.8612	0.101*	
C27	0.32148 (18)	0.91669 (17)	0.8667 (5)	0.0580 (16)	
C28	0.41337 (16)	0.82819 (17)	0.6946 (5)	0.0533 (15)	
C29	0.41773 (17)	0.81258 (18)	0.6102 (5)	0.0629 (17)	
H29	0.4191	0.8259	0.5541	0.075*	
C30	0.4200 (2)	0.7715 (2)	0.6235 (6)	0.080 (2)	
H30	0.4231	0.7525	0.5781	0.097*	
C31	0.4168 (2)	0.7659 (2)	0.7131 (6)	0.094 (2)	
H31	0.4172	0.7413	0.7407	0.112*	
C32	0.40934 (17)	0.86949 (18)	0.7240 (5)	0.0567 (16)	
C33	0.48763 (18)	0.90588 (19)	0.8414 (5)	0.0701 (18)	
H33	0.4788	0.8803	0.8331	0.084*	
C34	0.52802 (19)	0.9110 (2)	0.8497 (5)	0.0757 (19)	
H34	0.5454	0.8901	0.8461	0.091*	
C35	0.54161 (19)	0.9475 (2)	0.8621 (5)	0.0701 (18)	
H35	0.5684	0.9519	0.8658	0.084*	
C36	0.51533 (19)	0.97918 (19)	0.8692 (4)	0.0614 (16)	
C37	0.52598 (19)	1.0189 (2)	0.8857 (5)	0.0694 (17)	
H37	0.5524	1.0254	0.8909	0.083*	
C38	0.4990 (2)	1.0473 (2)	0.8937 (5)	0.0713 (18)	
H38	0.5072	1.0728	0.9054	0.086*	
C39	0.45792 (18)	1.03930 (18)	0.8848 (4)	0.0580 (15)	
C40	0.4279 (2)	1.06707 (18)	0.8917 (5)	0.0650 (17)	
H40	0.4339	1.0931	0.9048	0.078*	
C41	0.3902 (2)	1.05618 (19)	0.8798 (5)	0.0681 (17)	
H41	0.3703	1.0747	0.8840	0.082*	
C42	0.38101 (18)	1.01689 (18)	0.8599 (4)	0.0613 (16)	
H42	0.3550	1.0099	0.8509	0.074*	
C43	0.44656 (17)	1.00040 (17)	0.8670 (4)	0.0519 (14)	
C44	0.47548 (17)	0.97014 (18)	0.8604 (4)	0.0522 (14)	
C45	0.44756 (18)	0.7762 (3)	0.3692 (8)	0.217 (7)	
H45A	0.4706	0.7660	0.3986	0.325*	
H45B	0.4488	0.7710	0.3039	0.325*	
H45C	0.4248	0.7638	0.3948	0.325*	

### Atomic displacement parameters (Å^2^)

**Table:**

	*U*^11^	*U*^22^	*U*^33^	*U*^12^	*U*^13^	*U*^23^
Cu1	0.0515 (5)	0.0466 (4)	0.0721 (6)	0.0005 (3)	−0.0048 (4)	−0.0029 (4)
Cu2	0.0478 (4)	0.0464 (4)	0.0693 (6)	−0.0023 (3)	−0.0005 (4)	−0.0026 (4)
N1	0.046 (3)	0.051 (3)	0.061 (3)	0.009 (2)	−0.004 (2)	−0.001 (2)
N2	0.047 (3)	0.046 (3)	0.068 (4)	0.007 (2)	0.002 (2)	0.000 (2)
N3	0.047 (3)	0.050 (3)	0.079 (4)	0.005 (2)	0.001 (3)	−0.007 (3)
N4	0.043 (3)	0.051 (3)	0.063 (3)	0.001 (2)	−0.003 (2)	−0.002 (2)
O1	0.050 (3)	0.066 (3)	0.088 (4)	0.002 (2)	−0.002 (2)	0.004 (2)
O2	0.081 (3)	0.065 (3)	0.130 (5)	−0.015 (3)	−0.021 (3)	0.023 (3)
O3	0.060 (3)	0.075 (3)	0.140 (5)	−0.015 (3)	0.007 (3)	−0.005 (3)
O4	0.080 (3)	0.082 (3)	0.076 (4)	−0.001 (3)	−0.008 (3)	−0.005 (3)
O5	0.0933 (17)	0.0914 (17)	0.0919 (18)	−0.0022 (10)	−0.0005 (10)	0.0002 (10)
O6	0.096 (4)	0.110 (5)	0.130 (5)	0.002 (4)	−0.022 (4)	−0.021 (4)
O7	0.052 (2)	0.051 (2)	0.080 (3)	−0.003 (2)	−0.005 (2)	0.010 (2)
O8	0.069 (3)	0.084 (3)	0.094 (4)	−0.003 (3)	−0.004 (3)	0.032 (3)
O9	0.057 (3)	0.074 (3)	0.111 (4)	−0.007 (2)	0.010 (3)	0.013 (3)
O10	0.062 (3)	0.050 (3)	0.075 (3)	−0.005 (2)	0.004 (2)	−0.011 (2)
O11	0.098 (4)	0.048 (3)	0.081 (4)	−0.007 (2)	0.011 (3)	−0.003 (3)
O12	0.136 (5)	0.051 (3)	0.086 (4)	0.011 (3)	0.001 (3)	−0.002 (3)
O13	0.092 (3)	0.080 (3)	0.076 (3)	−0.011 (3)	−0.009 (3)	0.005 (3)
O14	0.095 (4)	0.115 (4)	0.091 (4)	−0.005 (3)	−0.001 (3)	−0.003 (3)
O15	0.45 (2)	0.300 (17)	0.37 (2)	−0.042 (16)	−0.035 (16)	−0.220 (17)
C1	0.059 (4)	0.051 (4)	0.080 (5)	−0.005 (3)	0.005 (4)	−0.017 (3)
C2	0.068 (4)	0.079 (5)	0.097 (5)	0.001 (4)	−0.007 (4)	−0.011 (4)
C3	0.070 (5)	0.098 (5)	0.112 (6)	0.020 (4)	−0.014 (4)	−0.019 (5)
C4	0.062 (5)	0.097 (6)	0.139 (7)	−0.003 (4)	0.000 (5)	−0.020 (5)
C5	0.057 (4)	0.053 (4)	0.091 (5)	−0.005 (3)	−0.007 (4)	−0.015 (4)
C6	0.053 (4)	0.051 (4)	0.133 (6)	−0.006 (3)	0.005 (4)	−0.028 (5)
C7	0.074 (5)	0.094 (5)	0.105 (6)	0.006 (4)	−0.003 (5)	−0.008 (5)
C8	0.085 (5)	0.119 (7)	0.134 (7)	−0.001 (5)	−0.010 (5)	−0.071 (6)
C9	0.098 (6)	0.063 (5)	0.183 (8)	−0.004 (4)	0.002 (6)	−0.026 (6)
C10	0.051 (4)	0.069 (4)	0.093 (5)	0.002 (3)	−0.005 (4)	0.014 (4)
C11	0.059 (4)	0.060 (4)	0.078 (4)	0.006 (3)	−0.004 (3)	−0.004 (3)
C12	0.054 (4)	0.075 (4)	0.076 (4)	0.013 (3)	−0.005 (3)	−0.006 (4)
C13	0.057 (4)	0.080 (4)	0.066 (4)	−0.006 (3)	−0.004 (3)	0.003 (3)
C14	0.057 (3)	0.065 (4)	0.051 (4)	−0.006 (3)	0.000 (3)	0.005 (3)
C15	0.070 (4)	0.069 (4)	0.067 (4)	−0.020 (3)	−0.002 (3)	0.004 (3)
C16	0.084 (4)	0.053 (3)	0.072 (4)	−0.013 (3)	0.003 (4)	−0.006 (3)
C17	0.072 (4)	0.050 (3)	0.059 (4)	−0.001 (3)	0.004 (3)	0.002 (3)
C18	0.086 (4)	0.053 (4)	0.081 (4)	0.000 (3)	0.001 (4)	−0.005 (3)
C19	0.087 (5)	0.059 (4)	0.086 (5)	0.022 (4)	0.004 (4)	−0.002 (4)
C20	0.064 (4)	0.064 (4)	0.079 (4)	0.007 (3)	0.002 (3)	−0.004 (3)
C21	0.057 (3)	0.047 (3)	0.046 (3)	−0.002 (3)	0.002 (3)	0.004 (3)
C22	0.055 (3)	0.048 (3)	0.044 (3)	0.000 (3)	−0.001 (3)	0.002 (3)
C23	0.052 (4)	0.053 (4)	0.068 (4)	−0.001 (3)	0.002 (3)	−0.008 (3)
C24	0.064 (4)	0.073 (4)	0.075 (5)	0.000 (3)	−0.008 (4)	−0.004 (4)
C25	0.068 (4)	0.093 (5)	0.086 (5)	0.015 (4)	−0.018 (4)	−0.007 (4)
C26	0.051 (4)	0.090 (5)	0.111 (6)	0.000 (4)	−0.008 (4)	−0.014 (5)
C27	0.060 (4)	0.039 (3)	0.075 (5)	−0.003 (3)	0.004 (3)	−0.001 (3)
C28	0.042 (3)	0.045 (3)	0.073 (4)	−0.001 (3)	0.001 (3)	−0.004 (3)
C29	0.057 (4)	0.057 (4)	0.074 (5)	−0.004 (3)	0.002 (3)	−0.010 (3)
C30	0.088 (5)	0.058 (4)	0.096 (5)	0.005 (4)	0.005 (4)	−0.019 (4)
C31	0.125 (6)	0.049 (4)	0.107 (6)	0.010 (4)	0.007 (5)	−0.003 (4)
C32	0.047 (3)	0.048 (4)	0.076 (5)	−0.005 (3)	0.004 (3)	−0.010 (3)
C33	0.058 (4)	0.062 (4)	0.091 (5)	0.000 (3)	0.002 (4)	−0.010 (3)
C34	0.052 (4)	0.081 (4)	0.094 (5)	0.016 (3)	0.001 (4)	−0.010 (4)
C35	0.049 (3)	0.087 (4)	0.074 (4)	0.002 (3)	0.001 (3)	0.001 (4)
C36	0.061 (4)	0.069 (4)	0.054 (4)	−0.011 (3)	−0.001 (3)	0.001 (3)
C37	0.056 (4)	0.077 (4)	0.076 (4)	−0.019 (3)	−0.004 (3)	0.006 (3)
C38	0.073 (4)	0.064 (4)	0.077 (4)	−0.023 (3)	0.001 (3)	0.001 (3)
C39	0.063 (4)	0.056 (3)	0.056 (4)	−0.011 (3)	0.004 (3)	0.006 (3)
C40	0.075 (4)	0.051 (3)	0.069 (4)	−0.004 (3)	−0.003 (3)	0.001 (3)
C41	0.073 (4)	0.058 (4)	0.073 (4)	0.012 (3)	0.001 (4)	0.006 (3)
C42	0.053 (3)	0.058 (4)	0.073 (4)	0.004 (3)	−0.004 (3)	0.001 (3)
C43	0.055 (3)	0.053 (3)	0.048 (3)	−0.003 (3)	−0.003 (3)	0.000 (3)
C44	0.049 (3)	0.058 (3)	0.049 (3)	−0.009 (3)	0.000 (3)	0.000 (3)
C45	0.199 (14)	0.35 (2)	0.097 (10)	−0.035 (15)	−0.029 (9)	0.080 (12)

### Geometric parameters (Å, °)

**Table:**

Cu1—O1	1.935 (4)	C12—C13	1.340 (8)
Cu1—N1	2.008 (5)	C12—H12	0.9300
Cu1—O4	2.012 (5)	C13—C14	1.412 (9)
Cu1—N2	2.017 (5)	C13—H13	0.9300
Cu1—O13	2.249 (5)	C14—C22	1.400 (8)
Cu2—O7	1.944 (4)	C14—C15	1.420 (8)
Cu2—O10	1.947 (4)	C15—C16	1.335 (9)
Cu2—N3	2.015 (5)	C15—H15A	0.9300
Cu2—N4	2.016 (5)	C16—C17	1.432 (9)
Cu2—O14	2.248 (5)	C16—H16	0.9300
N1—C11	1.321 (7)	C17—C21	1.390 (8)
N1—C22	1.360 (7)	C17—C18	1.410 (9)
N2—C20	1.329 (7)	C18—C19	1.343 (9)
N2—C21	1.367 (7)	C18—H18	0.9300
N3—C33	1.299 (7)	C19—C20	1.399 (9)
N3—C44	1.350 (7)	C19—H19	0.9300
N4—C42	1.333 (7)	C20—H20	0.9300
N4—C43	1.359 (7)	C21—C22	1.417 (8)
O1—C5	1.286 (8)	C23—C24	1.335 (8)
O2—C5	1.214 (8)	C23—C27	1.480 (8)
O3—C4	1.349 (9)	C24—C25	1.445 (9)
O3—C1	1.377 (7)	C24—H24	0.9300
O4—C10	1.196 (8)	C25—C26	1.312 (10)
O5—C10	1.249 (8)	C25—H25	0.9300
O6—C6	1.354 (9)	C26—H26	0.9300
O6—C9	1.379 (11)	C28—C29	1.339 (8)
O7—C27	1.283 (7)	C28—C32	1.478 (8)
O8—C27	1.228 (7)	C29—C30	1.415 (9)
O9—C26	1.359 (8)	C29—H29	0.9300
O9—C23	1.371 (7)	C30—C31	1.313 (10)
O10—C32	1.277 (7)	C30—H30	0.9300
O11—C32	1.233 (7)	C31—H31	0.9300
O12—C31	1.358 (8)	C33—C34	1.395 (9)
O12—C28	1.360 (7)	C33—H33	0.9300
O13—H13A	0.8500	C34—C35	1.340 (9)
O13—H13B	0.8501	C34—H34	0.9300
O14—H14A	0.8519	C35—C36	1.409 (9)
O14—H14B	0.8500	C35—H35	0.9300
O15—C45	1.301 (9)	C36—C44	1.401 (8)
O15—H15	0.8200	C36—C37	1.424 (9)
C1—C2	1.320 (9)	C37—C38	1.342 (9)
C1—C5	1.488 (9)	C37—H37	0.9300
C2—C3	1.451 (10)	C38—C39	1.435 (8)
C2—H2	0.9300	C38—H38	0.9300
C3—C4	1.319 (11)	C39—C40	1.400 (8)
C3—H3	0.9300	C39—C43	1.407 (8)
C4—H4	0.9300	C40—C41	1.348 (9)
C6—C7	1.257 (10)	C40—H40	0.9300
C6—C10	1.537 (10)	C41—C42	1.407 (8)
C7—C8	1.329 (10)	C41—H41	0.9300
C7—H7	0.9300	C42—H42	0.9300
C8—C9	1.309 (12)	C43—C44	1.432 (8)
C8—H8	0.9300	C45—H45A	0.9600
C9—H9	0.9300	C45—H45B	0.9600
C11—C12	1.393 (8)	C45—H45C	0.9600
C11—H11	0.9300		
			
O1—Cu1—N1	169.33 (19)	C15—C16—H16	119.3
O1—Cu1—O4	94.15 (18)	C17—C16—H16	119.4
N1—Cu1—O4	93.28 (19)	C21—C17—C18	116.5 (6)
O1—Cu1—N2	89.53 (18)	C21—C17—C16	118.5 (6)
N1—Cu1—N2	81.21 (19)	C18—C17—C16	125.1 (6)
O4—Cu1—N2	163.4 (2)	C19—C18—C17	119.7 (6)
O1—Cu1—O13	96.10 (18)	C19—C18—H18	120.2
N1—Cu1—O13	91.95 (18)	C17—C18—H18	120.1
O4—Cu1—O13	86.9 (2)	C18—C19—C20	120.9 (7)
N2—Cu1—O13	108.89 (18)	C18—C19—H19	119.7
O7—Cu2—O10	95.21 (16)	C20—C19—H19	119.4
O7—Cu2—N3	170.34 (19)	N2—C20—C19	121.3 (6)
O10—Cu2—N3	91.23 (18)	N2—C20—H20	119.2
O7—Cu2—N4	91.33 (17)	C19—C20—H20	119.5
O10—Cu2—N4	170.05 (19)	N2—C21—C17	123.7 (6)
N3—Cu2—N4	81.39 (18)	N2—C21—C22	116.0 (5)
O7—Cu2—O14	92.35 (18)	C17—C21—C22	120.4 (6)
O10—Cu2—O14	96.00 (19)	N1—C22—C14	123.8 (5)
N3—Cu2—O14	94.1 (2)	N1—C22—C21	116.4 (5)
N4—Cu2—O14	91.20 (19)	C14—C22—C21	119.8 (5)
C11—N1—C22	116.9 (5)	C24—C23—O9	110.2 (6)
C11—N1—Cu1	129.7 (4)	C24—C23—C27	133.2 (6)
C22—N1—Cu1	113.4 (4)	O9—C23—C27	116.7 (6)
C20—N2—C21	118.0 (5)	C23—C24—C25	106.0 (6)
C20—N2—Cu1	129.0 (4)	C23—C24—H24	127.1
C21—N2—Cu1	113.0 (4)	C25—C24—H24	126.8
C33—N3—C44	116.1 (5)	C26—C25—C24	106.2 (7)
C33—N3—Cu2	130.4 (4)	C26—C25—H25	126.7
C44—N3—Cu2	113.5 (4)	C24—C25—H25	127.1
C42—N4—C43	118.1 (5)	C25—C26—O9	111.5 (7)
C42—N4—Cu2	129.1 (4)	C25—C26—H26	124.4
C43—N4—Cu2	112.6 (4)	O9—C26—H26	124.1
C5—O1—Cu1	125.1 (4)	O8—C27—O7	127.3 (6)
C4—O3—C1	104.9 (6)	O8—C27—C23	118.5 (6)
C10—O4—Cu1	107.2 (5)	O7—C27—C23	114.2 (6)
C6—O6—C9	104.3 (8)	C29—C28—O12	111.1 (5)
C27—O7—Cu2	122.0 (4)	C29—C28—C32	130.6 (6)
C26—O9—C23	106.0 (6)	O12—C28—C32	118.3 (6)
C32—O10—Cu2	111.5 (4)	C28—C29—C30	106.0 (6)
C31—O12—C28	104.4 (6)	C28—C29—H29	127.2
Cu1—O13—H13A	87.5	C30—C29—H29	126.8
Cu1—O13—H13B	130.1	C31—C30—C29	106.0 (7)
H13A—O13—H13B	104.9	C31—C30—H30	127.0
Cu2—O14—H14A	77.5	C29—C30—H30	127.0
Cu2—O14—H14B	150.6	C30—C31—O12	112.5 (7)
H14A—O14—H14B	105.5	C30—C31—H31	123.8
C45—O15—H15	108.8	O12—C31—H31	123.7
C2—C1—O3	111.5 (6)	O11—C32—O10	123.7 (6)
C2—C1—C5	133.2 (7)	O11—C32—C28	120.1 (7)
O3—C1—C5	115.1 (6)	O10—C32—C28	116.2 (6)
C1—C2—C3	105.6 (7)	N3—C33—C34	125.4 (6)
C1—C2—H2	127.3	N3—C33—H33	117.9
C3—C2—H2	127.1	C34—C33—H33	116.6
C4—C3—C2	105.6 (7)	C35—C34—C33	118.1 (6)
C4—C3—H3	127.3	C35—C34—H34	120.1
C2—C3—H3	127.1	C33—C34—H34	121.9
C3—C4—O3	112.4 (8)	C34—C35—C36	120.2 (6)
C3—C4—H4	123.6	C34—C35—H35	120.0
O3—C4—H4	124.0	C36—C35—H35	119.8
O2—C5—O1	127.4 (6)	C44—C36—C35	116.3 (6)
O2—C5—C1	119.7 (7)	C44—C36—C37	118.2 (6)
O1—C5—C1	112.9 (7)	C35—C36—C37	125.5 (6)
C7—C6—O6	107.8 (7)	C38—C37—C36	121.9 (6)
C7—C6—C10	138.6 (9)	C38—C37—H37	119.0
O6—C6—C10	113.3 (8)	C36—C37—H37	119.1
C6—C7—C8	113.3 (9)	C37—C38—C39	121.6 (6)
C6—C7—H7	122.0	C37—C38—H38	119.1
C8—C7—H7	124.5	C39—C38—H38	119.2
C9—C8—C7	104.5 (9)	C40—C39—C43	116.8 (6)
C9—C8—H8	127.5	C40—C39—C38	125.5 (6)
C7—C8—H8	128.0	C43—C39—C38	117.8 (6)
C8—C9—O6	109.9 (8)	C41—C40—C39	120.1 (6)
C8—C9—H9	124.3	C41—C40—H40	119.9
O6—C9—H9	125.8	C39—C40—H40	119.9
O4—C10—O5	125.9 (8)	C40—C41—C42	120.1 (6)
O4—C10—C6	121.1 (7)	C40—C41—H41	120.0
O5—C10—C6	113.0 (8)	C42—C41—H41	119.9
N1—C11—C12	123.6 (6)	N4—C42—C41	121.6 (6)
N1—C11—H11	118.3	N4—C42—H42	119.1
C12—C11—H11	118.2	C41—C42—H42	119.2
C13—C12—C11	119.3 (6)	N4—C43—C39	123.2 (5)
C13—C12—H12	120.1	N4—C43—C44	116.6 (5)
C11—C12—H12	120.6	C39—C43—C44	120.2 (5)
C12—C13—C14	120.5 (6)	N3—C44—C36	123.9 (6)
C12—C13—H13	119.9	N3—C44—C43	115.8 (5)
C14—C13—H13	119.7	C36—C44—C43	120.3 (6)
C22—C14—C13	116.0 (6)	O15—C45—H45A	108.1
C22—C14—C15	118.8 (6)	O15—C45—H45B	104.1
C13—C14—C15	125.2 (6)	H45A—C45—H45B	109.5
C16—C15—C14	121.2 (6)	O15—C45—H45C	116.0
C16—C15—H15A	119.4	H45A—C45—H45C	109.5
C14—C15—H15A	119.3	H45B—C45—H45C	109.5
C15—C16—C17	121.3 (6)		

### Hydrogen-bond geometry (Å, °)

**Table:**

*D*—H···*A*	*D*—H	H···*A*	*D*···*A*	*D*—H···*A*
O14—H14B···O5^i^	0.85	1.76	2.611 (8)	177
O14—H14A···O8	0.85	2.07	2.634 (7)	124
O13—H13B···O11	0.85	1.96	2.749 (7)	154
O13—H13A···O2	0.85	1.87	2.708 (7)	169
O15—H15···O4	0.82	1.86	2.474 (17)	131

Symmetry codes: (i) *x*, *y*, *z*+1.
